# Utilizing artificial intelligence to assess academic exam anxiety, perceived stress, and achievement motivation among college students

**DOI:** 10.3389/fpsyt.2026.1686106

**Published:** 2026-03-09

**Authors:** Zuhal Y. Hamd, Zamzam A. Mohmed, Nouf Alroqaiba, Sherine Mohamed Elzagawy, Hala Abd Ellatif Elsayed, Maha Mahmoud Lashin, Maha Aldera, Amal I. Alorainy

**Affiliations:** 1 Department of Radiological Sciences, College of Health and Rehabilitation Sciences, Princess Nourah bint Abdulrahman University, Riyadh, Saudi Arabia; 2 Department of Health Sciences, College of Health and Rehabilitation Sciences, Princess Nourah bint Abdulrahman University, Riyadh, Saudi Arabia; 3 Department of Biomedical Engineering, College of Engineering, Princess Nourah bint Abdulrahman University, Riyadh, Saudi Arabia; 4 Department of Communication Sciences, College of Health and Rehabilitation Sciences, Princess Nourah bint Abdulrahman University, Riyadh, Saudi Arabia

**Keywords:** academic anxiety, achievement motivation, artificial intelligence, fuzzy system, perceived stress

## Abstract

**Introduction:**

Exam anxiety is a multidimensional construct combining physiological reactions and affective responses that can hinder academic performance. Academic stress reflects students’ perceived pressure related to workload, deadlines, and self-evaluation. Achievement motivation refers to students’ drive to attain optimal performance. This study evaluated academic anxiety, perceived stress, and achievement motivation before and after examinations and examined whether artificial intelligence can effectively assess students’ psychological states.

**Methods:**

A cross-sectional, repeated-measures design was used. Academic institution students completed an online questionnaire assessing exam anxiety, perceived academic stress, and achievement motivation before and after examinations. Data were analysed using SPSS for statistical modelling. In parallel, a fuzzy logic system (FLS) was developed to model students’ psychological states and estimate exam anxiety and achievement motivation in relation to perceived stress. Outputs from SPSS and FLS were compared to evaluate concordance.

**Results:**

SPSS analysis showed a significant interaction between perceived stress and achievement motivation prior to examinations (b = 0.02, 95% CI: 0.01–0.02, p < 0.001). This moderating effect was not observed after examinations (b = 0.00, 95% CI: −0.01–0.01, p = 0.554). The FLS results were consistent with conventional statistical findings, demonstrating strong agreement in identifying levels of exam anxiety and the role of achievement motivation before exams.

**Discussion:**

Achievement motivation moderates the relationship between perceived stress and exam anxiety only in the pre-examination period, highlighting the temporal nature of this interaction. The alignment between SPSS and FLS outcomes suggests that artificial intelligence, particularly fuzzy logic systems, can efficiently evaluate students’ academic exam anxiety. These findings support the potential use of AI-based tools for psychological state assessment in educational settings, especially for early identification of students at risk of heightened exam anxiety.

## 1 Introduction

This section will explained in detail the relationship between academic exam anxiety, perceived stress, and achievement motivation and their effect on students before and after the exam. Also, a fuzzy system, a type of artificial intelligence, will be used to measure the effect of perceived stress and achievement motivation on students’ academic exam anxiety.

### 1.1 Relationship between perceived stress and achievement motivation

Students experience stress throughout their college years since they are immersed in an unfamiliar social and academic setting (1). Motivation for achievement as a sign of academic stress in students was studied by Kaur and Kaur (2), who discovered a detrimental relationship between academic success and school-related stress. At this point in their education, stress is one of the most prevalent issues for university students, particularly throughout the transition from high school to college. How they manage stress is mirrored in their drive for achievement (3). College students’ perceived stress is adversely affected by shifts in their learning styles, obstacles in communication, and diminished employment opportunities.

Achievement motivation as a psychological trait pushes individuals to meet their internalized standards of excellence. A behavior is achievement-motivated if it includes “competition with a standard of excellence (4). According to empirical research, university students experience challenges adjusting to their new learning environment and controlling their motivation for achievement (5, 6). Motivation indirectly predicted academic outcomes through stress appraisal (7), and stress negatively predicted motivation (8).

The degree of students’ motivation may significantly affect their ability to discover a coping mechanism while dealing with stress (9). Numerous psychologists have shown that people vary in how hard they strive for achievement. Intense fears of failure that result in high stress levels can impede performance and outcomes. Still, moderate levels of fear of failure that create moderate stress levels can enhance performance and results (10).

### 1.2 Relationship between perceived stress and academic anxiety

“Anxiety” is a maladaptive level of “stress” (11). Anxiety, a psychological and physiological state, is a normal stress response. Still, prolonged exposure and higher levels of perceived stress may lead to adverse consequences, including the development of anxiety disorder (12). Anxiety is a common psychological experience that contributes to individual differences in psychological states (13). Although stress and anxiety are frequently used synonymously, there is one distinction between stress and anxiety. Stress may respond to a threat in a situation, and anxiety is responsive to stress. Students’ perceptions of stress played a moderating effect in the relationship between stressors and anxiety symptoms (14). When students are anxious during their studies, they often perform worse overall. They cannot achieve a high-quality total performance, making them think they cannot meet the demands of the situation that are greater than their abilities. This perception of stress then intensifies, which ultimately leads to psychological distress in the individual (15). It has been demonstrated that situational stress contributes to increased state anxiety in students, most likely because of their awareness of being evaluated and having their accomplishments compared to those of others. Students with anxiety tendencies are more likely to suffer state anxiety in stressful settings, as evidenced by the fact that highly trait-anxious students frequently have more state anxiety than low trait-anxious students.

### 1.3 The relationship between academic anxiety and achievement motivation

Anxiety is an adverse motivational condition that manifests in circumstances where a person feels highly threatened (16). While a moderate amount of anxiety is natural, extreme anxiety can pose a significant threat. Over time, academic anxiety might have additional adverse effects; over specific academic assignments, anxiety may rise as a student’s performance in class declines (17). Additionally, anxiety during exams or assessments can have adverse effects on one’s ability to advance academically via the educational system. While it is usual for students to have anxiety before an exam, it can cause problems when it gets out of control (18).

College students who exhibit signs of academic anxiety are likely to be students who either experience anxiety or who are at a higher risk of developing it in the future (11). Anxiety has an impact on motivation and performance; high rates of motivation are among students with an average value of situational anxiety, and high-performing students have a medium level of anxiety. Potential and academic performance have been found to differ significantly. Personality theories suggest this gap can be attributed to personality traits, particularly anxiety and achievement motivation (19). Several variables may mediate the relationship between students’ achievement motivation and stress and anxiety levels (20).

### 1.4 Relationship between perceived stress, academic anxiety, and achievement motivation

An increasing body of research indicates that anxiety and performance are related. Anxiety is associated with performance deficits (16). Test anxiety and academic success had an overall association of −0.29, according to a meta-analysis based on hundreds of researches (21). There have been numerous attempts to provide a theoretical explanation for the adverse effects of anxiety on performance.

Alam (22) studied the relationship between stress, test anxiety, and achievement; the results showed that there was a negative correlation between stress, test anxiety, and student achievement. There is a significant relationship between student’s stress and anxiety on achievement motivation.

The goal of the development of Attentional Control Theory (ACT) (23) is to elucidate the relationship between anxiety and motivation (24). There is an abundance of data linking anxiety—whether viewed as an emotional state or a personality trait—to performance deficits in a variety of activities (16).

In compliance with ACT, Stressful situations cause trait-anxious people to focus more intently on worried, task-irrelevant thoughts.

ACT predicts that people with high anxiety would often identify and respond to threat-related cues more quickly than people with low anxiety. They will also be more likely to view ambiguous stimuli as potentially hazardous. Consistent with ACT’s prediction that increased distractibility and a consequent drop in attentional controls as a result of anxiety, several studies on anxiety and cognition have attained the same result: high levels of stress are linked to poor task performance, particularly in intellectually taxing activities (25).

Test anxiety is a widespread problem for college students, affecting their academic performance. In psychology, self-report surveys may be biased and arbitrary. Unlike prior methods, artificial intelligence objectively assesses intellectual test anxiety, tension, and motivation among college students. This work creates an AI-powered tool to objectively examine psychological factors that influence academic success, therefore enhancing educational psychology and technology. This study aims to elucidate academic exam anxiety, perceived stress, and desire for achievement. This research creates tailored treatments and support systems based on students’ psychological profiles to enhance mental health and academic success.

Although AI-driven approaches to investigate psychological aspects influencing academic achievement are limited, prior studies have explored this domain. Conventional methods such as self-report questionnaires may underreport psychological distress owing to social desirability bias. This project will develop and evaluate an AI-driven instrument to measure academic exam anxiety, perceived stress, and motivation for achievement. This study has the potential to revolutionize the identification and treatment of psychiatric disorders in college students via the use of AI. Moreover, various approaches have been presented in literature about the use of machine learning models (26–31).

Fuzzy logic is beneficial for capturing subjective, complex, and imprecise concepts such as mental states, but it has limitations.

Expert judgments and subjective assessments are usually used to establish membership functions, which may be biased and inconsistent. Specialists may define membership functions differently based on their mental state ideas. Many cultural, social, and personal influences affect human psychology. Fuzzy logic models may misrepresent these details. Complex fuzzy logic models may be computationally expensive with large datasets and fuzzy sets. Researchers may use fuzzy logic with machine learning to improve model performance and overcome these limits. They can create membership functions and build rule bases methodically.

## 2 Methods

### 2.1 Study setting

This study was a cross-sectional study conducted among undergraduate college students at academic institution during May 2023–2024 after getting approval from the Princess Nourah bint Abdulrahman University (PNU)IRB committee Log No: 22–0998 using the well-structured questionnaire survey form to evaluate stress and anxiety in college students before and after the final exam.

This study investigated the mediation effect of perceived stress and achievement motivation on academic anxiety among college students during final exams. A total of 389 undergraduate students recruited from academic institution participated in the study. This is a cross-sectional study. All the students selected from various courses at PNU were considered the population of the study. The students were chosen randomly from academic institution students. In the frame of the study, two mediation models were tested by utilizing the SPSS. Perceived stress and achievement motivation are expected to contribute to the variance of academic anxiety.

### 2.2 Data instruments

#### 2.2.1 Demographic information form

Students will also complete a short demographic questionnaire that includes age, academic program, college, and year (level) in college.

It will be conducted with college students.

#### 2.2.2 Perceived stress scale

The Perceived stress scale (PSS) is the most widely used psychological instrument for measuring the perception of stress (15). The Cronbach’s alpha of the PSS-10 was evaluated at >.70 in all 12 studies in which it was used. The test-retest reliability of the PSS-10 was assessed in four studies and met the criterion of >.70 in all cases. According to Cohen’s Laboratory for the Study of Stress, Immunity, and Disease (2021), the PSS is currently translated into 25 languages other than English.

#### 2.2.3 Revised achievement motives scale-10

(R-AMS) is a well-established and frequently used scale to assess hope of success and fear of failure (32). A revised 10-item version (AMS-R) fits the theoretically intended two-factor model. The adequate fit could be validated in cross-validation procedures. Furthermore, the revised scales provided adequate reliability, lower interscale correlations, and criterion-related validity concerning typical criteria of achievement-related behavior. (33: PsycINFO Database Record (c) 2012 APA).

#### 2.2.4 Academic anxiety scale

Eleven 4-point Likert-type questions were created to capture a variety of anxieties and concerns related to academic settings and circumstances. The response options (“1 Not at all typical of me,” “2 Somewhat typical of me,” “3 Quite typical of me,” and “4 Very typical of me”) were the same as those on the CTAR. According to Cassady, J.C., Pierson, E. E., & Starling, J. M. (34), the fundamental objective of this scale was to create a simple, broad contextual representation for concerns encountered in academic contexts. The authors reported the scale’s outstanding internal consistency (α =.90). Additionally, the CFA showed that the model fit was good: 2 (33, N = 260) = 70.02, p <.001; ϵ2/df = 2.12; RMSEA = .059; RMSEA 90% CI [.04; 08]; CFI = 0.97; TLI = 0.96; NFI = 0.96; SRMR = 0.037. Using Cronbach’s alpha, an excellent internal.

### 2.3 Ethical consideration

The study approved by The Institutional Review Board (IRB) at Princess Nourah bint Abdulrahman University (PNU) (Log No.22-0998).

### 2.4 Analysis of academic anxiety questionnaire

The analysis was used SPSS and fuzzy systems (artificial intelligence) as examples of different approaches that can be used in the academic anxiety field.

#### 2.4.1 Statistical analysis

Statistical analysis was carried out using SPSS 27. We used frequencies and percentages to present categorical variables, whereas continuous variables were expressed as the median and interquartile range (IQR). The internal consistency of domains was investigated using Cronbach’s alpha. A one-sample Wilcoxon signed-rank test was used to assess the difference in scores between pre- and post-exam periods. The differences in scores based on students’ study levels and colleges were assessed using a Kruskal-Wallis rank sum test. The bivariate correlations between scores were assessed using a Spearman correlation analysis. The magnitude of the correlation was interpreted as weak (R = 0.20 to 0.30), moderate (R = 0.3 to 0.6), vital (R = 0.6 to 0.8), and very strong (R = 0.8 to 0.99).Statistical significance was set at p < 0.05.

#### 2.4.2 Fuzzy system

**Figure 1:**
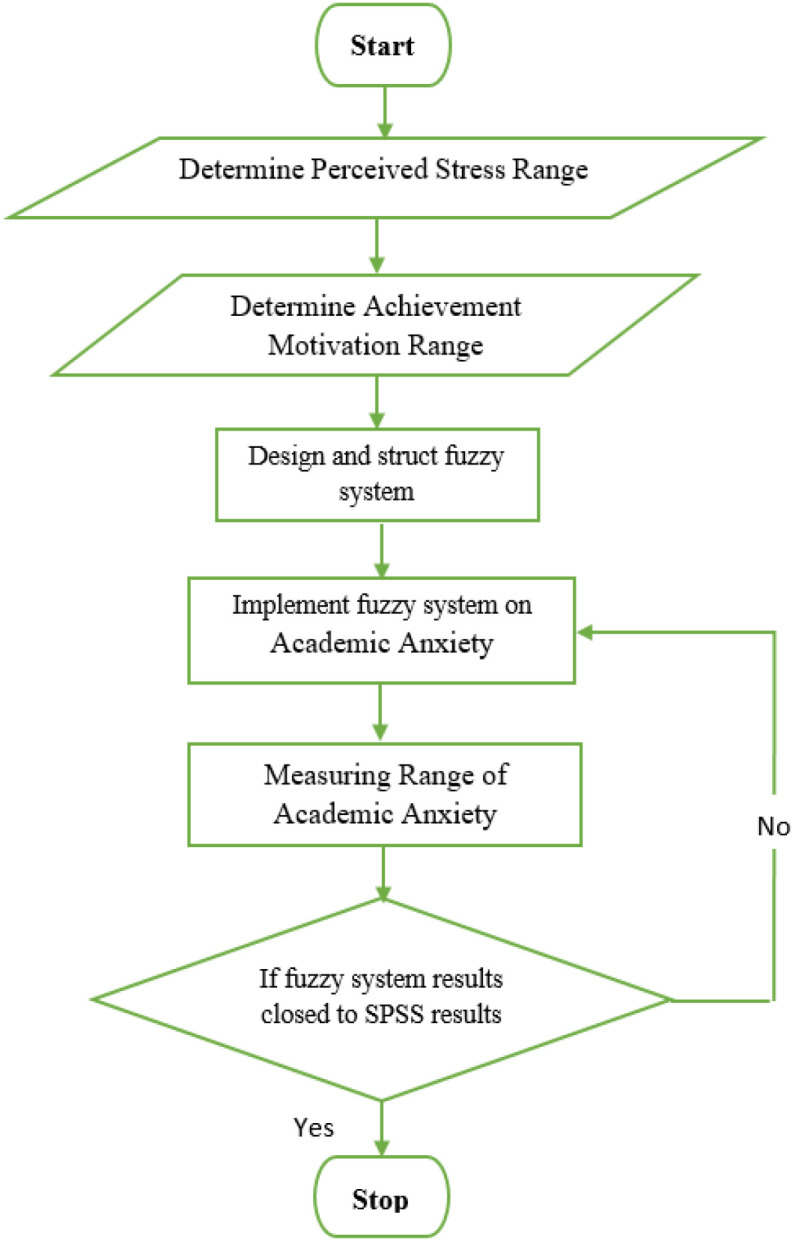
Flow chart of artificial intelligence of academic anxiety system.

A Fuzzy logic system is used as a type of artificial intelligence to measure and analyze the effect of perceived stress and achievement motivation on students’ academic exam anxiety. The flow chart of the academic anxiety fuzzy system explains in detail the steps of collecting data, designing the fuzzy system, and implementing that system on the collected data, as shown in **Figure 1**.

##### 2.4.2.1 Measurement students’ academic exam anxiety via fuzzy logic system

A fuzzy system is an artificial intelligence system based on the mathematical system for analyzing analog input values in continuous logic on discrete values of either 1 or 0 (true or false, respectively). Input, processing, and output stages are part of the Fuzzy system. The input stage includes membership functions known as fuzzy sets for converting the crisp input values to a fuzzy value through fuzzification. Each appropriate rule is invoked by the processing stage, which produces a result for each and then combines the rules’ outcomes. Finally, the output stage transforms the combined outcome back into a particular output value of the control through defuzzification (35).

##### 2.4.2.2 Fuzzy system architecture

**Figure 2:**
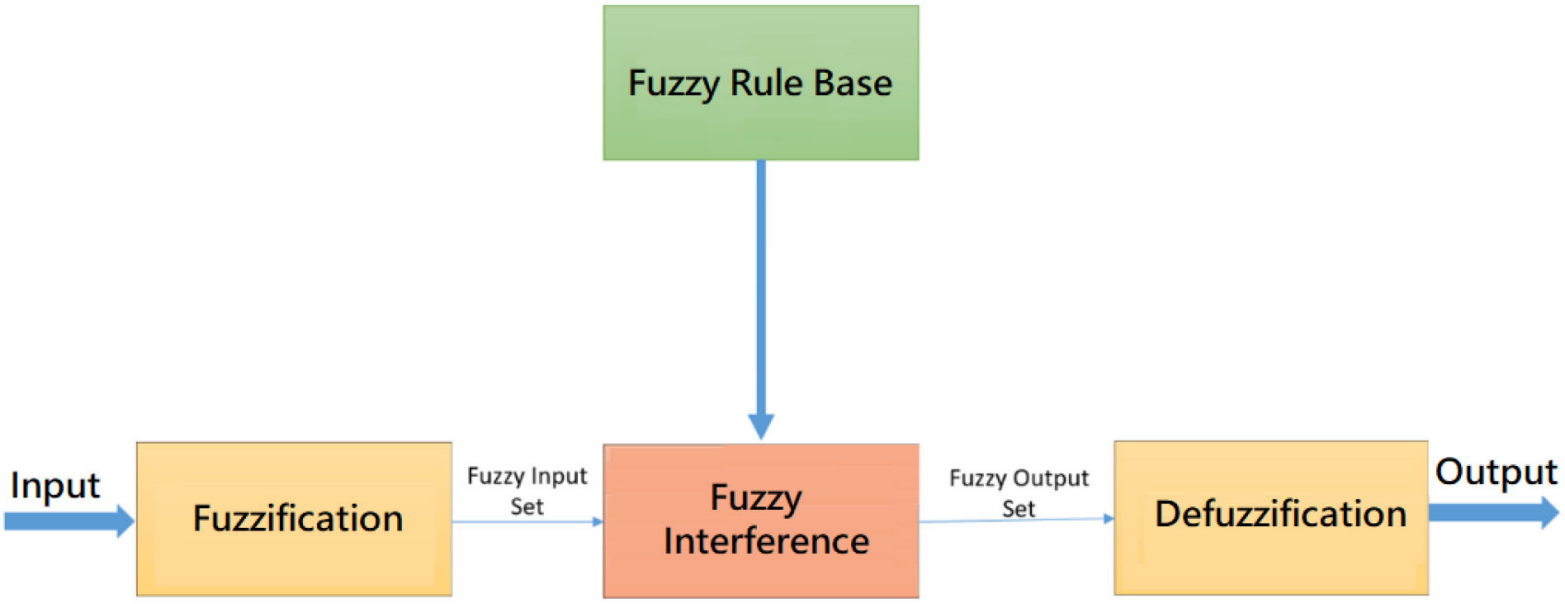
Fuzzy system structure.

Fuzzy systems can handle problems with imprecise and incomplete data or that are complex; furthermore, such systems can also model nonlinear functions of arbitrary sets. A fuzzifier (fuzzification) and a demulsifier (defuzzification), If-Then rules (fuzzy rule base), and an inference engine are the main components of fuzzy systems (36), as shown in **Figure 2**.

In this work, a fuzzy system was designed to measure and analyze the effect of perceived stress and achievement motivation on students’ academic exam anxiety.

A fuzzy system with two inputs (perceived stress and achievement motivation) and one output (academic anxiety) was designed and implemented based on the questionnaire results applied to the students of Princess Nourah University (PNU).

**Figure 3:**
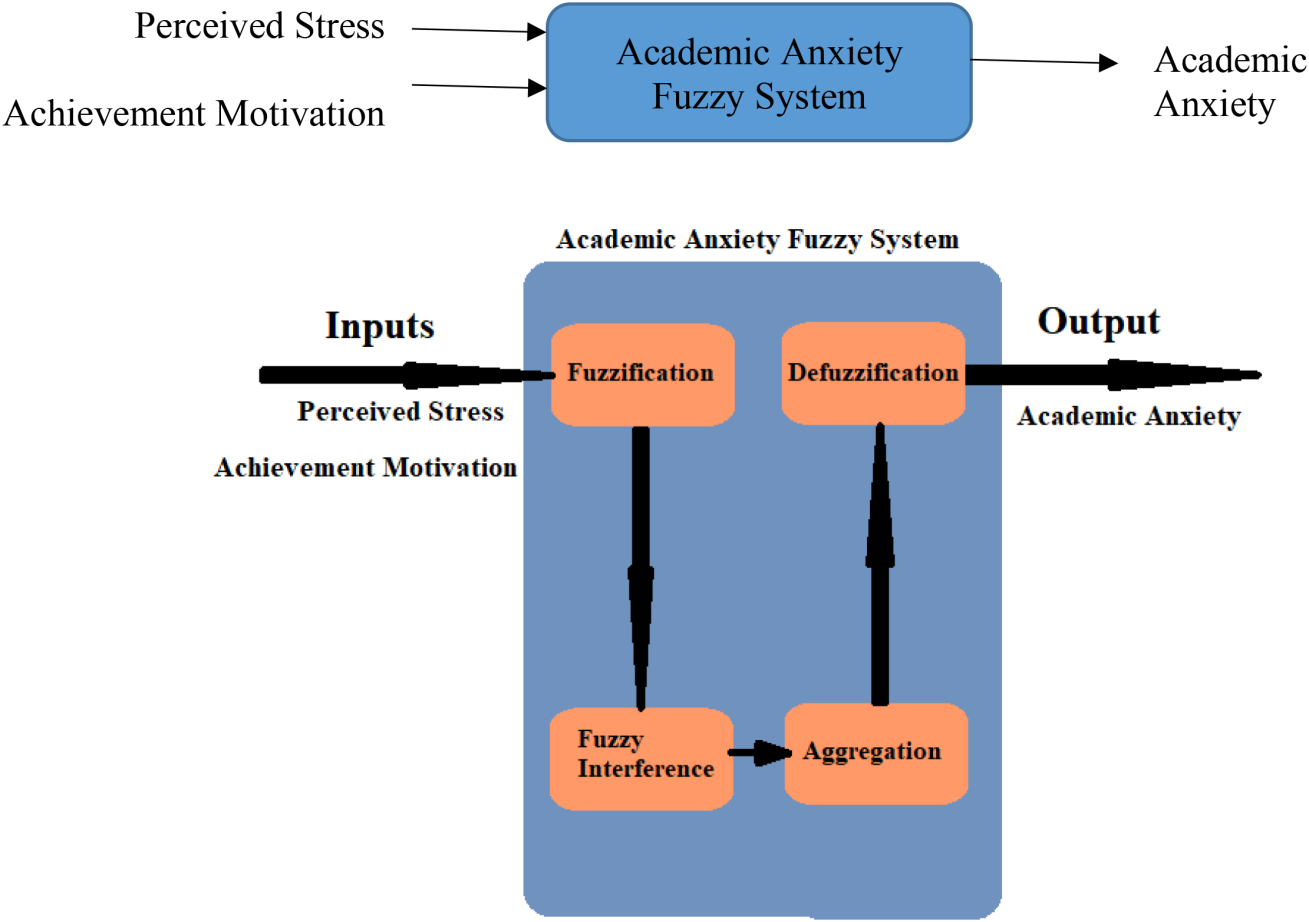
Academic anxiety fuzzy system.

Inputs, output, fuzzification, and defuzzification steps of our academic anxiety fuzzy system appear in **Figure 3**.

The fuzzy logic system was built using MATLAB software. Fuzzification, defuzzification, and If-Then rules are the main processes in fuzzy logic systems (37), as shown in **Figure 3**.

The first step in the fuzzification process involves converting the input data (affected variables) into a fuzzy set via fuzzy linguistic variables and membership functions (triangular membership functions with low, medium, and high parameters).

##### 2.4.2.3 Inputs – output membership functions of academic anxiety fuzzy system

**Figure 4:**
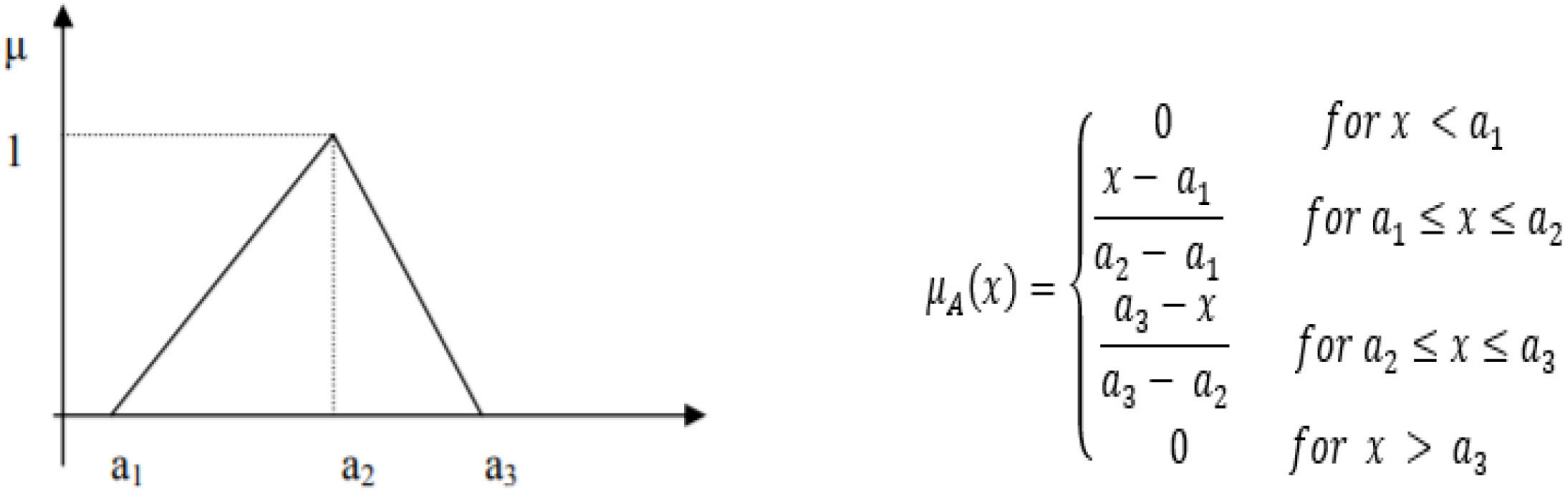
Triangular membership function [4].

**Figure 5:**
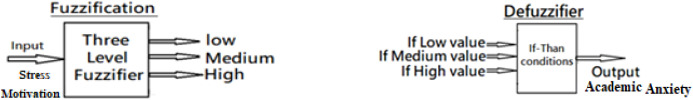
Fuzzification and defuzzification (membership function) process.

For points a1, a2, and a3, the triangular membership function, as shown in **Figure 4** in fuzzy system A = (a1, a2, a3), represented an increasing function (a1 to a2) and decreasing function (a2 to a3) that can write as;

The triangular membership function of academic anxiety fuzzy systems is used to fuzzify inputs (perceived stress and achievement motivation) in three values of fuzzy sets (Low, Medium, and High) and defuzzify the output (academic anxiety), as shown in **Figure 5**.

**Figure 6:**
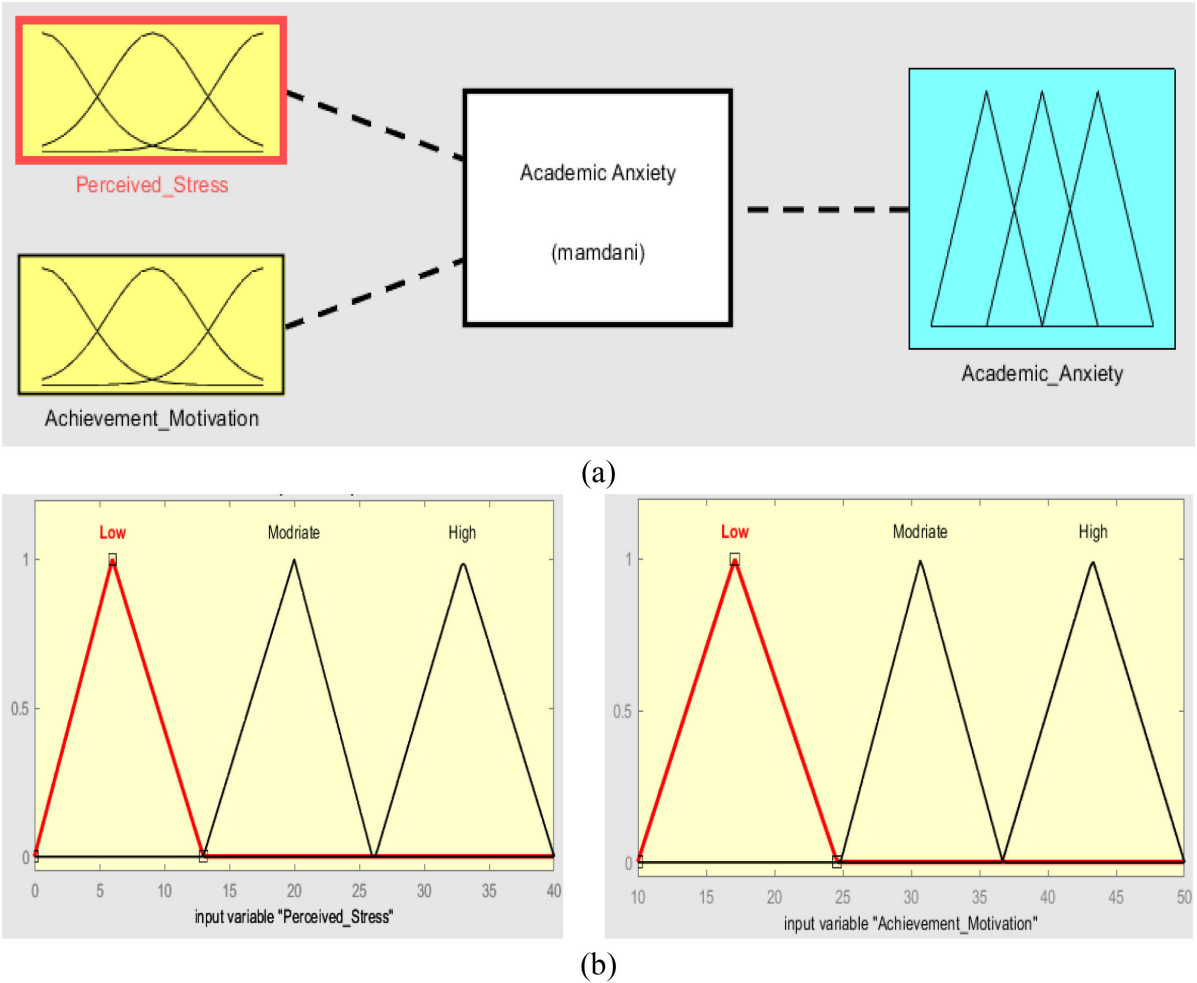
**(A)** Academic anxiety fuzzy system, **(B)** Input variables (in triangular membership function).

Triangular membership function fuzzification is the input and output of fuzzy systems, as shown in **Figures 6a, b**.

**Table 1** shows the values of the academic anxiety fuzzy system output for the three adopted ranges. It explains the fuzzy systems inputs’ low, medium, and high levels (perceived stress and achievement motivation).

**Table 1:** Membership functions of fuzzy system output.

Fuzzy system inputs variables	Membership function used	Range of inputs		
		Low	Medium	High
Perceived Stress	Triangular MF	0 - 13	13 - 26	26 - 40
Achievement Motivation	Triangular MF	10 - 25	25 - 36	36 - 50

##### 2.4.2.4 If – then rules of academic anxiety fuzzy system

**Figure 7:**
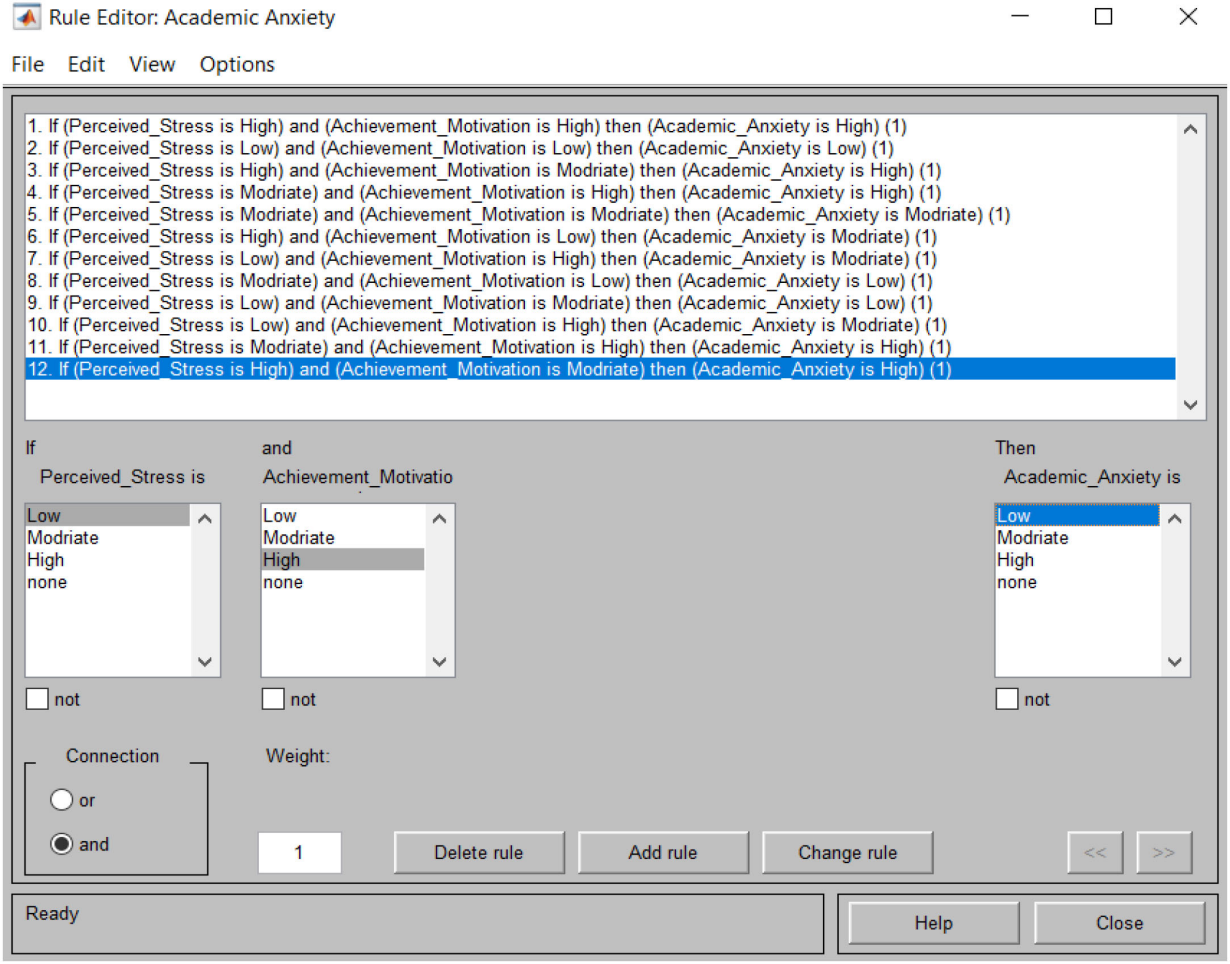
If-then rules used in academic anxiety fuzzy system.

Fuzzy system rules, or—Then rules, are used to join the inputs and outputs of the designed systems. In the presented academic anxiety fuzzy system, the fuzzy rules explain changes in academic anxiety as a result of changes in perceived stress and achievement motivation, as shown in **Table 2**. The inference process is carried out via If-Then rules, which link the impact of inputs to output, as shown in **Figure 7**.

**Table 2:** Perceived stress and achievement motivation values.

Output variables		Input variables	
Academic anxiety	Maximum value Minimum values	Perceived stress	Achievement motivation
		33.4	35.8
		5.3	15.5

##### 2.4.2.5 Maximum and minimize students’ academic exam anxiety values

**Figure 8:**
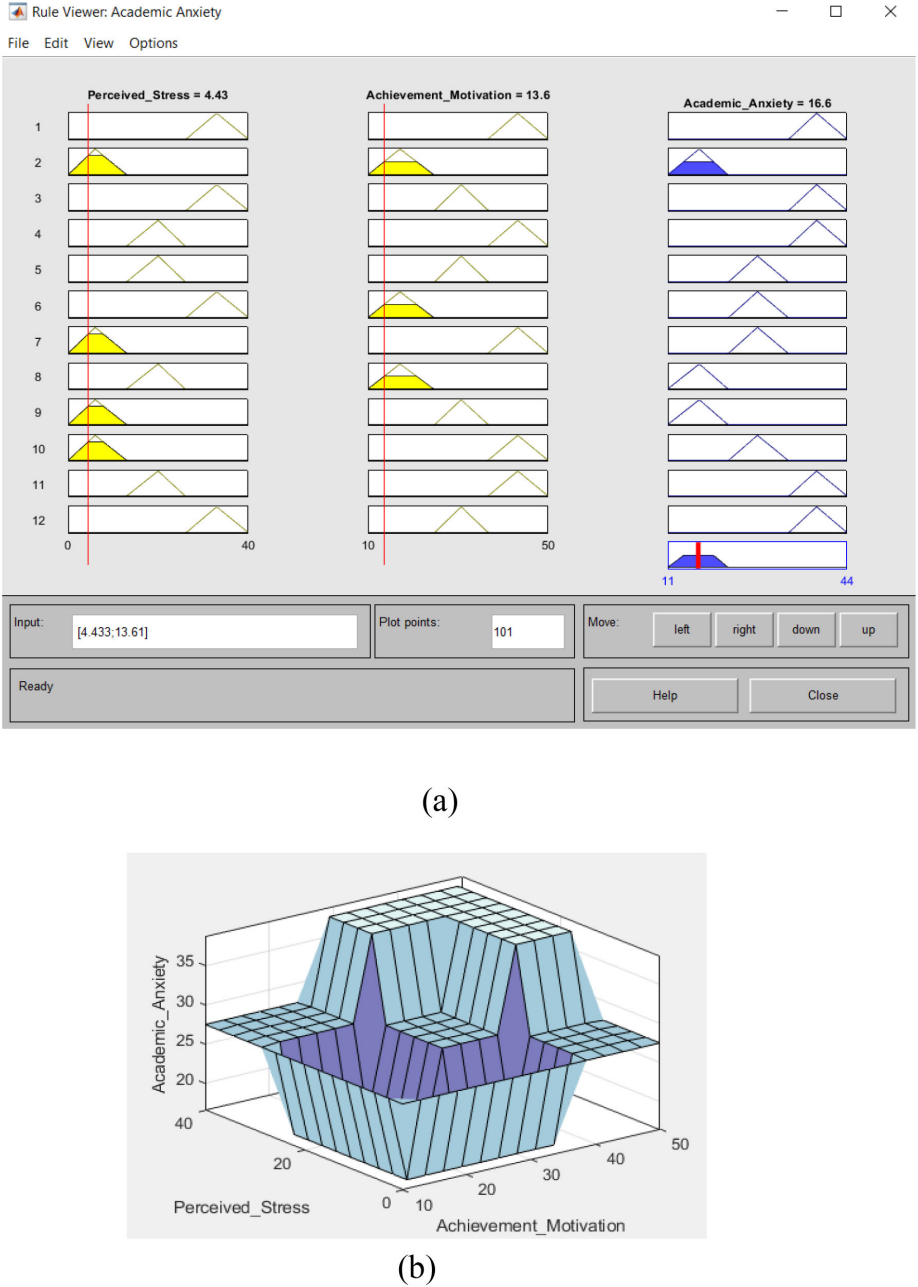
Academic anxiety fuzzy system **(A)** rules viewer of fuzzy system **(B)** surface viewer values.

The results of the academic anxiety fuzzy system designed to be implemented on perceived stress and achievement motivation samples are shown in **Table 2**. The maximum and minimum values of students’ academic exam anxiety appear in **Figures 8A, B**.

**Figure 9:**
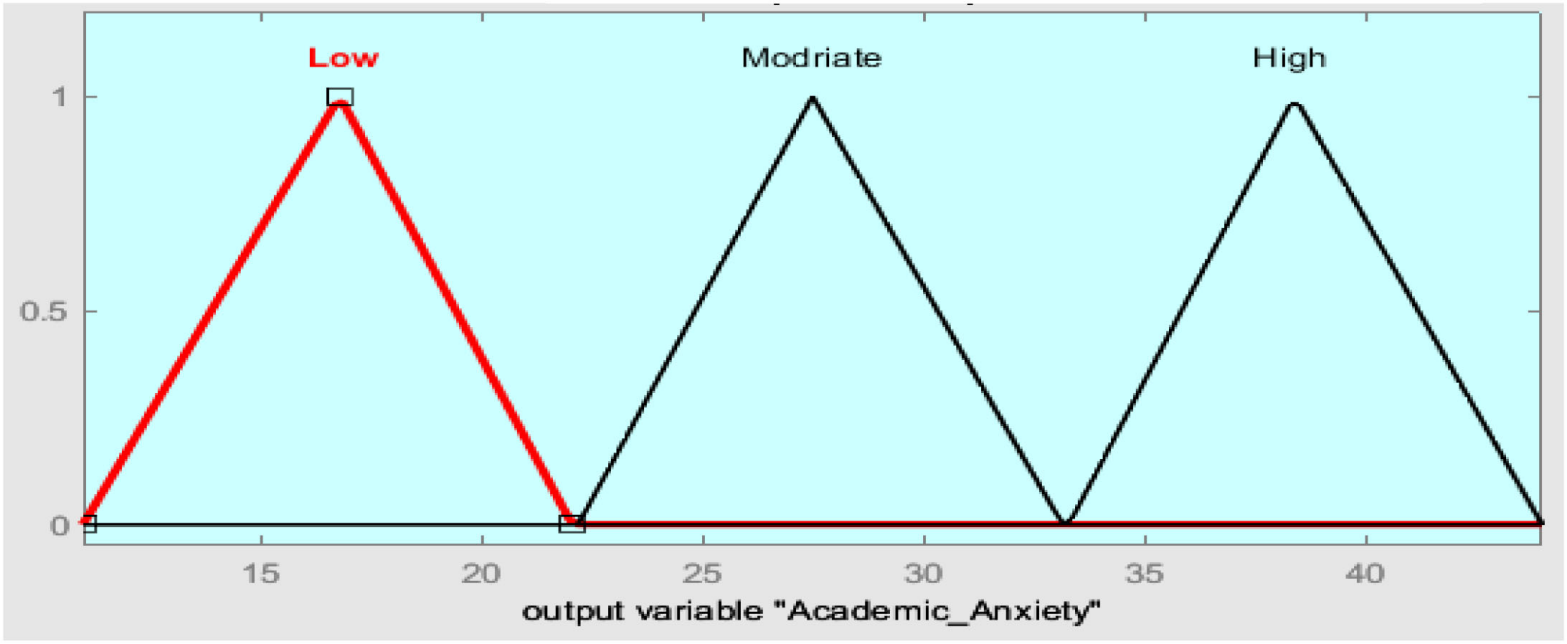
Output variable in triangular membership function.

Lastly, the defuzzification step is implemented, and the process is completed using membership functions to obtain students’ academic exam anxiety values, as in **Figure 9**.

#### 2.4.3 Proposed model architecture

**Figure 10:**
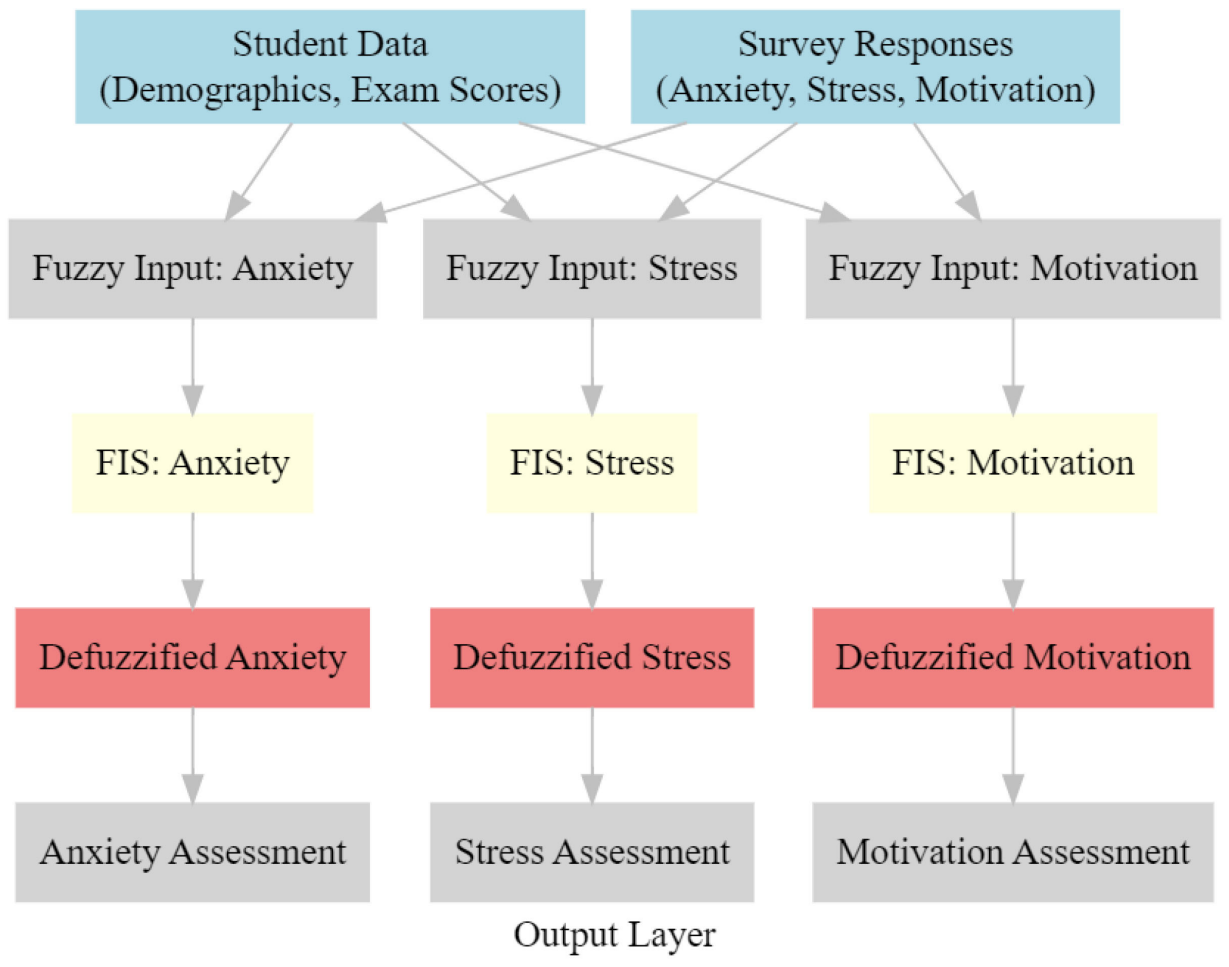
Architecture of the proposed model.

**Figure 10** presents the architecture of the proposed model. It consists of 5 layers.

##### 2.4.3.1 Input layer

It represents the data that is sent in. This data may include student demographics, test results, and survey responses.

##### 2.4.3.2 Fuzzification

The technique of fuzzification involves the transformation of crisp input data into fuzzy collections.

##### 2.4.3.3 The fuzzy inference system

It is a system that use fuzzy rules in order to analyze the levels of concern, stress, and motivation that an individual is experiencing.

##### 2.4.3.4 Defuzzification

The process of transforming fuzzy output into values that are clear is referred to as defuzzification since it is the process.

##### 2.4.3.5 Output layer

Evaluations of anxiety, tension, and motivation are provided by output layer. One may say that it is a representation of the results that the artificial intelligence model produced.

## 3 Results and discussion

The results of the current study showed that, before the exam, there was a substantial, moderate link between perceived stress and accomplishment motivation (R = 0.474, p < 0.001) and that this correlation was weaker in the post-exam period (R = 0.116, p = 0.023). Rama, it has been noted that college students who experience high-stress levels are likely to score worse on the accomplishment motivation scale (125.25) than those who experience low-stress levels (132.25). However, students with moderate stress levels exhibit significant motivation for accomplishment (146.41). The difference between the mean scores is statistically significant, as evidenced by the obtained F value (6.52), which is significant at the 5% level since the p-value of 0.003 is less than 0.05.

**Table 3** shows that the records of 389 students were analyzed in the current study. Most participants in the study were in the beginner level, comprising 55.5% (N = 216) of the sample, followed by intermediate-level students at 28.0% (N = 109). Clinical psychology emerged as the predominant specialty among students, with 32.9% (N = 60) of participants specializing in this area, followed by epidemiology (12.3%, N = 45).

**Table 3:** Academic characteristics of students.

Characteristic	N (%)
Study level	
Beginner (Levels 1-5)	216 (55.5%)
Intermediate (Levels 6-9)	109 (28.0%)
Seniors (Levels 10 and above)	64 (16.5%)
College	
College of Health and Rehabilitation Sciences	389 (100%)
Specialty	
Clinical Nutrition	42 (10.0%)
Occupational therapy	40
Clinical psychology	60 (32.9%)
Diagnostic radiology	40 (9.0%)
Epidemiology	45 (12.3%)
Health education	24 (6.2%)
Nuclear medicine	13 (0.8%)
Radiation therapy	20 (0.8%)
Respiratory Therapy	16 (0.8%)
Audiology	13 (8.2%)
Speech	15(0.3%)
Ultrasound	11 (2.8%)
Physiotherapy	50 (0.8%)

**Table 4** presents the descriptive statistics and **Table 5** presents the changes in scores of the different domains across different academic characteristics.

**Table 4:** Descriptive statistics of the main domains under study.

Characteristic	Median (IQR)	Mean ± SD	Min - Max	Cronbach Alpha
PSS before	24.0 (21.0 - 26.0)	23.2 ± 5.8	0.0 - 40.0	0.640
PSS after	18.0 (16.0 - 22.0)	18.8 ± 4.8	0.0 - 35.0	0.555
AMS before	36.0 (31.0 - 40.0)	35.6 ± 7.0	10.0 - 50.0	0.777
AMS after	20.0 (18.0 - 22.0)	20.0 ± 3.2	11.0 - 32.0	0.496
AAS before	25.0 (18.0 - 32.0)	25.7 ± 8.8	11.0 - 44.0	0.912
AAS after	26.0 (18.0 - 32.0)	25.7 ± 8.6	11.0 - 44.0	0.904

AAS, Academic anxiety scale; AMS, Achievement motivation scale; PSS, perceived stress scale.

**Table 5:** The changes in scores of the different domains across different academic characteristics. .

Characteristic	PSS		AMS		AAS	
	Median (IQR)	P-value	Median (IQR)	P-value	Median (IQR)	P-value
Study level		<0.001		<0.001		0.416
Beginner	0.0 (-7.0, 0.0)		-16.0 (-20.0, -8.0)		0.0 (-8.0, 9.3)	
Intermediate	-8.0 (-10.0, -2.0)		-19.0 (-23.0, -13.0)		-2.0 (-9.0, 9.0)	
Seniors	-1.5 (-8.3, 0.0)		-15.0 (-22.3, -10.0)		-2.0 (-8.0, 5.0)	
College		0.264		0.081		0.694
College of Health and Rehabilitation Sciences	-3.0 (-9.0, 0.0)		-16.5 (-22.0, -10.0)		-1.0 (-8.0, 8.3)	

Kruskal-Wallis rank sum test.

In this paper we ensure that the data used to train and test the model is accurate, clean, and representative. The proposed model avoids overfitting by using a parsimonious model with a minimal number of rules and membership functions. By monitoring the model’s performance over time and make necessary adjustments to maintain its reliability and validity.

In **Table 5** additionally, no significant differences were found in the changes in scores across colleges for PSS (p = 0.264), AMS (p = 0.081), and AAS (p = 0.694).

**Table 6** shows that moderation analysis was performed by constructing two multivariable generalized linear models using pre- and post-exam AAS scores as dependent variables (each variable in a separate model). The independent variables included students’ study levels, colleges, and the interaction terms of PSS * AMS. Results showed that the interaction effect of perceived stress and achievement motivation was significant before exams (β = 0.02, 95% CI: 0.01 to 0.02, p < 0.001) but not after exams (β = 0.00, 95% CI: -0.01 to 0.01, p = 0.554), indicating a moderating influence of achievement motivation on the relationship between perceived stress and academic anxiety in the pre-exam period exclusively.

**Table 6:** Predictors of academic anxiety before and after exams.

Characteristic	Before exam			After exam		
	Beta	95% CI*^1^*	P-value	Beta	95% CI*^1^*	P-value
Study level						
Beginner	Reference	Reference		Reference	Reference???	
Intermediate	0.15	-1.59, 1.89	0.863	1.91	-0.09, 3.90	0.062
Seniors	1.04	-1.00, 3.08	0.317	-0.06	-2.47, 2.35	0.961
College						
College of Health and Rehabilitation Sciences	Reference	Reference		Reference	Reference???	
PSS * AMS	0.02	0.01, 0.02	<0.001	0.00	-0.01, 0.01	0.554

**Figure 11:**
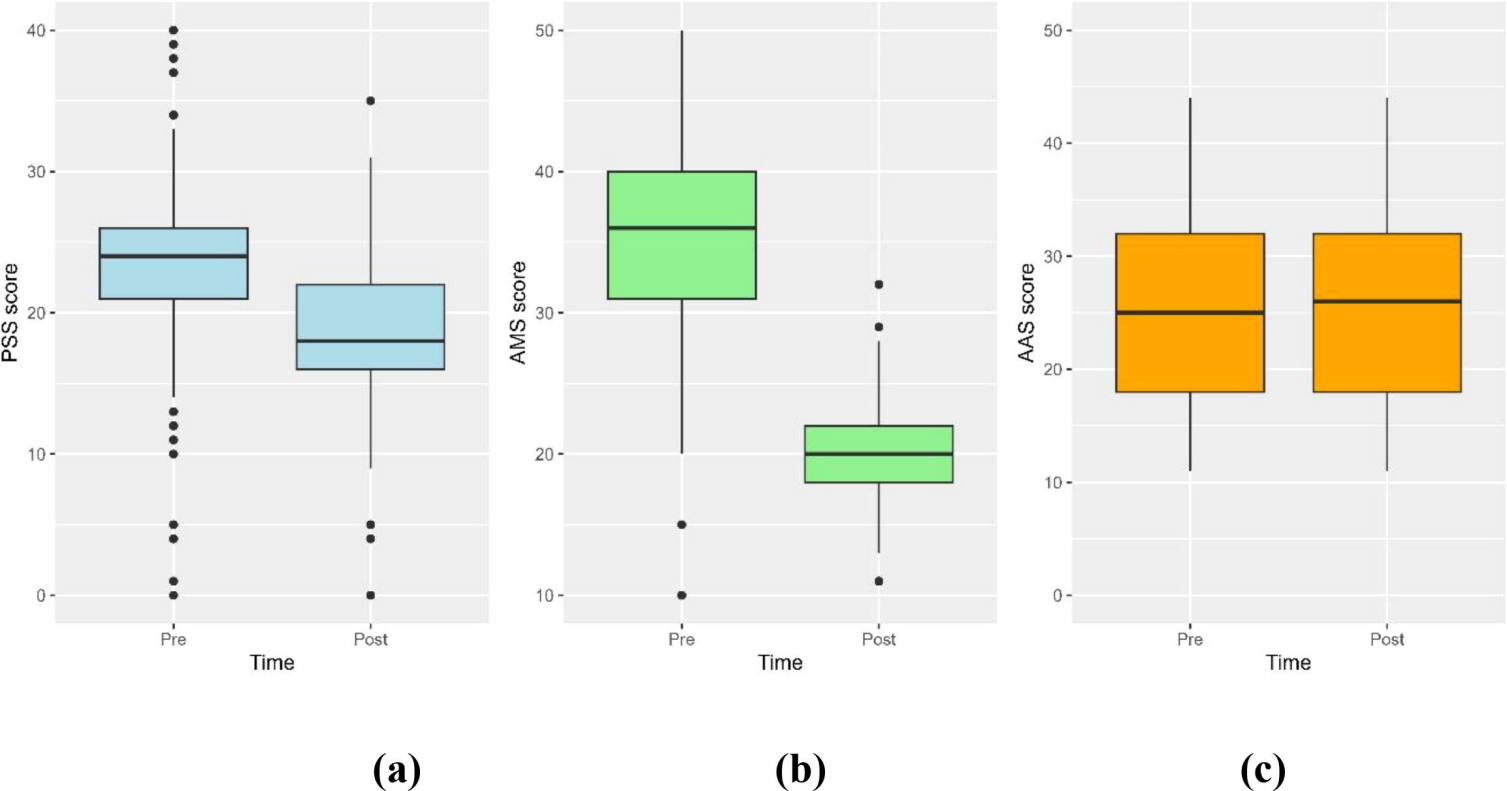
Three box plots comparing scores before and after an exam. Panel **(a)** shows PSS scores decreasing from around 23 to 18. Panel **(b)** illustrates AMS scores decreasing from about 35 to 20. Panel **(c)** shows stable AAS scores around 25 for both periods. Each plot indicates score distribution with whiskers and outliers.

*An asterisk indicates an interaction effect of moderation.

(**Figure 11A**). There was a significant difference between the pre-and post-exam periods (p < 0.001,.Similarly, the AMS score decreased significantly from the pre-exam period (median = 36.0, IQR, 31.0 to 40.0) to the post-exam period (median = 20.0, IQR, 18.0 to 22.0) with a p-value of < 0.001 (**Figure 11B**). The median (IQR) AAS scores were 25.0 (18.0 - 32.0) before the exam and 26.0 (18.0 - 32.0) after the exam, with no significant differences between the two periods (p = 0.846, **Figure 11C**).

**Figure 12:**
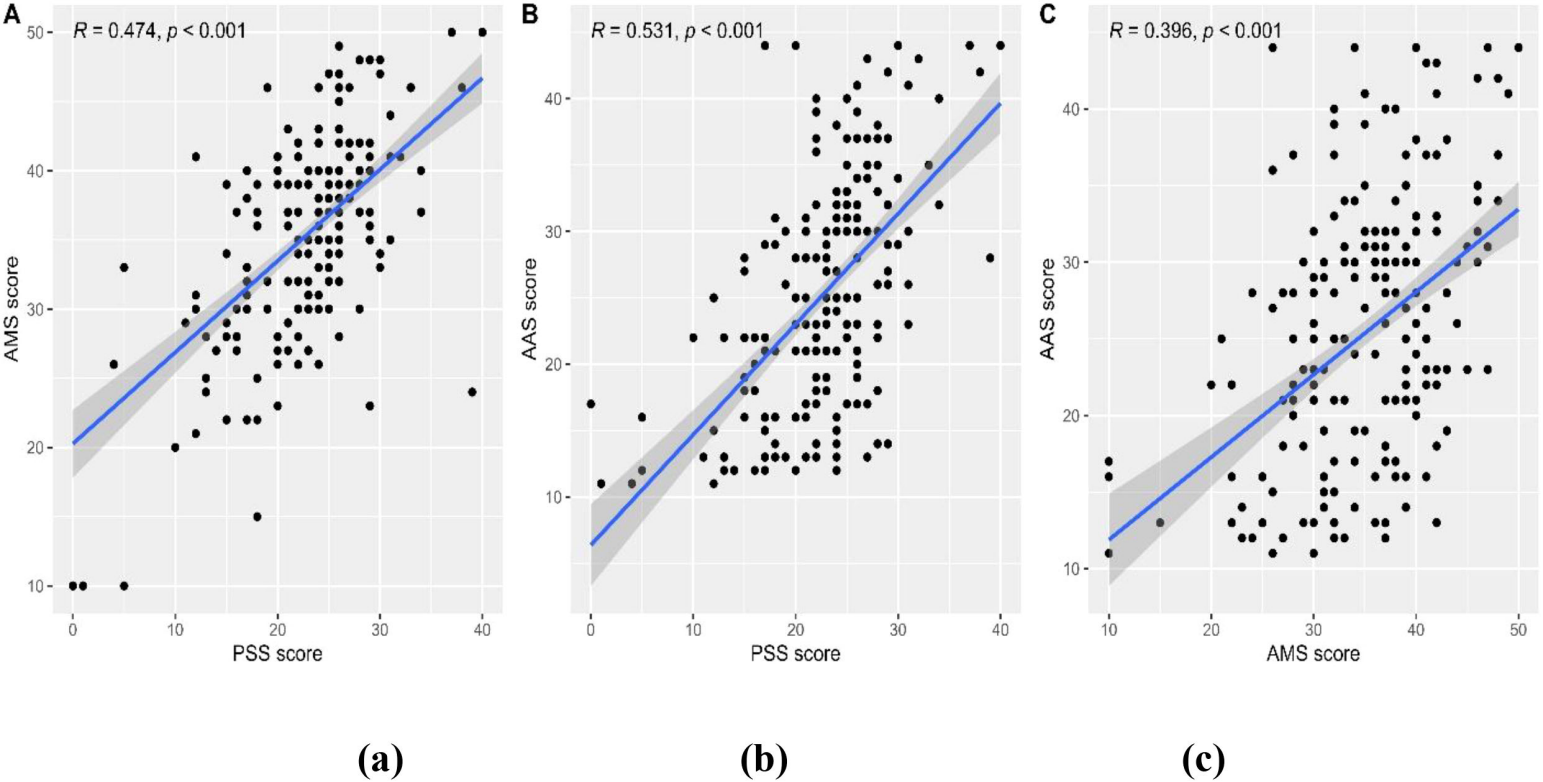
Scatter plots depict correlations with regression lines and R values. Plot **(A)** AMS score vs. PSS score (R = 0.474). Plot **(B)** AAS score vs. PSS score (R = 0.531). Plot **(C)** AAS score vs. AMS score (R = 0.396). All with p-values less than 0.001.

In general, there was a significant moderate correlation between perceived stress and achievement motivation before the exam (R = 0.474, p < 0.001, **Figure 12A**), and the correlation was weaker in the post-exam period (R = 0.116, p = 0.023, **Figure 12A**). Academic anxiety was also significantly and moderately correlated with the perceived stress in the pre-exam period (R = 0.531, p < 0.001, **Figure 12B**). Still, the correlation was no longer significant in the post-exam period (R = -0.063, p = 0.216, **Figure 12B**). Similarly, the correlation between academic anxiety and achievement motivation was moderate in the pre-exam period (R = 0.396, p < 0.001, **Figure 12C**) and non-significant in the post-exam period (R = 0.011, p = 0.833, **Figure 12C**).

**Figure 13:**
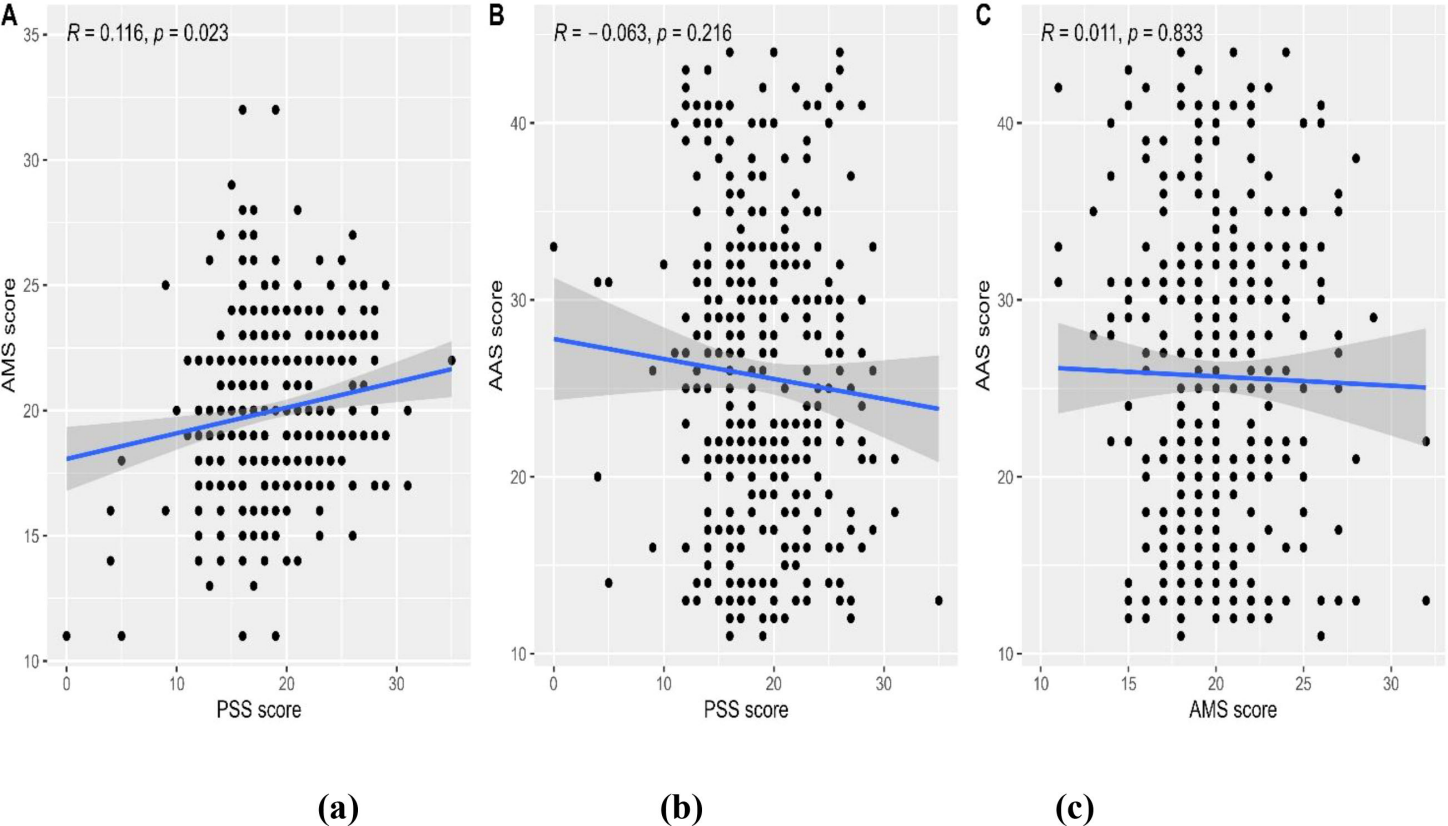
Three scatter plots labeled **(A–C)** display relationships between different scores. Plot **(A)** shows a slight positive correlation between AMS and PSS scores with R equals 0.116 and p equals 0.023. Plot **(B)** displays a slight negative correlation between AAS and PSS scores with R equals -0.063 and p equals 0.216. Plot **(C)** shows a negligible correlation between AAS and AMS scores with R equals 0.011 and p equals 0.833. Each plot features a blue trend line with a gray confidence interval.

**Figure 13A**). Academic anxiety was also significantly and moderately correlated with the perceived stress in the pre-exam period (R = 0.531, p < 0.001, **Figure 13B**). Still, the correlation was no longer significant in the post-exam period (R = -0.063, p = 0.216, **Figure 13B**). Similarly, the correlation between academic anxiety and achievement motivation was moderate in the pre-exam period (R = 0.396, p < 0.001, **Figure 13C**) and non-significant in the post-exam period (R = 0.011, p = 0.833, **Figure 13C**).

The test anxiety arises during examinations and is influenced by a person’s personality, cognition, and other characteristics. Anxiety is the primary psychological component of this condition, which is also characterized by various emotional arousal and physiological symptoms (38). Test anxiety has detrimental effects on students’ academic performance and mental health, including poor academic performance and academic difficulties. It also increases the risk of anxiety and depression (39). Long-term exam anxiety, however, can have adverse effects on people’s bodies, including sleep difficulties.

The current investigation discovered a substantial correlation between academic anxiety and psychological stress. Additionally, there was a significant and moderate correlation between academic anxiety and perceived stress during the pre-exam period (R = 0.531, p < 0.001). Additionally, according to Alam (22), there is a substantial positive association between test anxiety and academic stress for both the whole and sub-samples, suggesting that higher levels of academic stress are associated with higher levels of test anxiety and vice versa.

The result of the present study is supported by the findings of research done by Bankston and Zhou (40). They found a significant positive relationship between stress and the academic performance of college students. Also, Pavithra Narasimhan 2018 stated that a similar result of our study, which showed Self – concept (138.290 ± 20.7750) and Achievement motivation (30.530 ± 7.3601) at the same time controlling for students’ academic stress (82.360 ± 14.8851), which is enormously significant (r = 0.269, p-value < 0.01) levels. Regarding academic anxiety and achievement motivation, the current study revealed that the correlation between academic anxiety and achievement motivation was moderate in the pre-exam period (R = 0.396, p < 0.001) and non-significant in the post-exam period (R = 0.011, p = 0.833), shows that 53% of variance is explained by the regression model which shows that test anxiety affects students’ achievement in pre and post-test. Academic anxiety has a significantly negative relationship with achievement motivation, but its relationship with academic achievement was statistically insignificant.

They were concerned with the relationship between perceived stress and academic anxiety on students’ motivation for achievement. The present study found that Academic anxiety was also significantly and moderately correlated with the perceived stress in the pre-exam period similarly, the correlation between academic anxiety and achievement motivation was moderate in the pre-exam period (R = 0.396, p < 0.001, and non-significant in the post-exam period (R = 0.011, p = 0.833), this result in agreement with study of Ramaprabou and Dash (41) found that high levels of achievement motivation for students with moderate stress and less motivation for students with slight stress and with high-stress levels. Hence, an optimal moderate stress level seems beneficial for high academic motivation. The findings indicate that there were no significant interactions between the factors. For instance, no significant interaction F(14, 146)=.91, p=.56 between the dependent variable, stress, and the variables academic year and motivation. The same outcomes were observed for every other variable in this model. Hence, it can be inferred that although there were relationships between the distant variables—stress, motivation, and performance—no additional significant interactions were found. Additionally, the same study discovered that the data in this study meet the prerequisites for a mediator analysis. All three variables—achievement, motivation, and felt stress—were considerable.

Prior academic success and socio-economic status (SES) may affect academic study outcomes. These factors may conceal the independent-dependent relationship in findings. With more resources, tutors, and technology, wealthier students may do well academically. Academically successful students are more self-confident and set higher goals. Covariate analysis may reduce the influence of confounding variables in statistical models. Autonomous subgroup analyses, incorporating the confounding variable, may reveal group-specific effects. When adapting the Perceived Stress Scale (PSS) and the Received Achievement Motives Scale (R-AMS) for a specific cultural context, several concerns arise regarding their appropriateness. We ensure the constructs measured by the scales are understood and interpreted similarly across cultures. We translate the items accurately and ensuring that the translated items convey the same meaning as the original items. We ensure that the factor structure of the scales is the same across cultures. The original scales may be culturally biased, reflecting the values and norms of the culture in which they were developed.

Furthermore, this study demonstrated that stress and motivation are significantly correlated. Although there is little research on the subject, universities may find value in this conclusion regarding the connection between stress and motivation. Of course, there are various reasons why students could be inspired to study. Thus, it could be worthwhile to concentrate on this issue in future research. If stress stems from a lack of motivation, there might be a chance to provide specialized classes that assist pupils in regaining their motivation.

The degree to which a student experiences academic anxiety and perceived stress may be strongly impacted by demographic features among the student population. Comparatively, females are more likely to report experiencing greater levels of academic anxiety and stress than men (9). It is possible that this occurred as a result of biological causes, gender roles, and cultural expectations. There is a possibility that males may face academic stress; nevertheless, they may be less inclined to seek assistance or communicate their emotions, which may result in possible mental health problems. Younger students may be more susceptible to experiencing greater levels of stress as a result of the unfamiliarity of the academic environment and the additional pressure to adjust to new problems. The academic anxiety that older students experience may be exacerbated by extra pressures such as the responsibility they have toward their families, their professional commitments, and their financial worries.

Accuracy of AI models depends on the volume and quality of training data. Should the data be distorted or insufficient, the model would fail. Personality, society, and coping strategies might all affect how one interprets judgments created by AI. This study suggests that AI might objectively and properly identify academic test anxiety, perceived stress, and accomplishment incentive. AI helps us to identify students who could be struggling academically and act before things spirals out of control. We have to consider the ethical problems this technology generates as well as its limits.

## 4 Conclusion and future work

In this paper, anxiety among students of academic institution exams was measured through a questionnaire that was prepared and distributed to university students. Questionnaire results were analyzed using two techniques, SPSS and fuzzy system, to measure the effect of perceived stress and achievement motivation on students’ academic exam anxiety. A fuzzy system (as a type of artificial intelligence) was designed and implemented on the questionnaire results for the same purpose as SPSS. The results of the techniques used were compared to determine effectiveness and accuracy. The results prove the effectiveness and sufficiency of the proposed artificial intelligence, especially the fuzzy system, in the study of the mental health of university students. Future studies should look at the long-term effects of AI-based therapies; the AI model should be refined and the dataset should be enlarged. Likewise important is the development of open ethical guidelines for the development and use of artificial intelligence in educational environments.

## Data Availability

The original contributions presented in the study are included in the article/supplementary material. Further inquiries can be directed to the corresponding authors.
