# Correction: Utilizing artificial intelligence to assess academic exam anxiety, perceived stress, and achievement motivation among college students

**DOI:** 10.3389/fpsyt.2026.1830610

**Published:** 2026-07-07

**Authors:** Zuhal Y. Hamd, Zamzam A. Mohmed, Nouf Alroqaiba, Sherine Mohamed Elzagawy, Hala Abd Ellatif Elsayed, Maha Mahmoud Lashin, Maha Aldera, Amal I. Alorainy

**Affiliations:** 1 Department of Radiological Sciences, College of Health and Rehabilitation Sciences, Princess Nourah bint Abdulrahman University, Riyadh, Saudi Arabia; 2 Department of Health Sciences, College of Health and Rehabilitation Sciences, Princess Nourah bint Abdulrahman University, Riyadh, Saudi Arabia; 3 Department of Biomedical Engineering, College of Engineering, Princess Nourah bint Abdulrahman University, Riyadh, Saudi Arabia; 4 Department of Communication Sciences, College of Health and Rehabilitation Sciences, Princess Nourah bint Abdulrahman University, Riyadh, Saudi Arabia

**Keywords:** academic anxiety, achievement motivation, artificial intelligence, fuzzy system, perceived stress

All affiliations erroneously contained the abbreviation “PNU”. The abbreviation has been removed.

The **Acknowledgments** statement was erroneously omitted. The statement should be:

“This research was funded by the Deanship of Scientific Research at Princess Nourah bint Abdulrahman University, through the Research Funding Program, Grant No. (FRP-1444-37).”

The original version of this article has been updated.

